# HOXA3 Modulates Injury-Induced Mobilization and Recruitment of Bone Marrow-Derived Cells

**DOI:** 10.1002/stem.90

**Published:** 2009-07

**Authors:** Kimberly A Mace, Terry E Restivo, John L Rinn, Agnes C Paquet, Howard Y Chang, David M Young, Nancy J Boudreau

**Affiliations:** 1 Department of Surgery, University of California San FranciscoSan Francisco, California, USA; 2 Healing Foundation Centre, Faculty of Life Sciences, University of ManchesterManchester, United Kingdom; 3 Program in Epithelial Biology, Stanford University School of MedicineStanford, California, USA; 4 Department of Medicine, University of California San FranciscoSan Francisco, California, USA

**Keywords:** Wound repair, HOXA3, Bone marrow-derived cells, Endothelial progenitor cells, Inflammation, Angiogenesis, Vasculogenesis, GFP chimeras

## Abstract

The regulated recruitment and differentiation of multipotent bone marrow-derived cells (BMDCs) to sites of injury are critical for efficient wound healing. Previously we demonstrated that sustained expression of *HOXA3* both accelerated wound healing and promoted angiogenesis in diabetic mice. In this study, we have used green fluorescent protein-positive bone marrow chimeras to investigate the effect of *HOXA3* expression on recruitment of BMDCs to wounds. We hypothesized that the enhanced neovascularization induced by *HOXA3* is due to enhanced mobilization, recruitment, and/or differentiation of BMDCs. Here we show that diabetic mice treated with *HOXA3* displayed a significant increase in both mobilization and recruitment of endothelial progenitor cells compared with control mice. Importantly, we also found that *HOXA3*-treated mice had significantly fewer inflammatory cells recruited to the wound compared with control mice. Microarray analyses of *HOXA3*-treated wounds revealed that indeed HOXA3 locally increased expression of genes that selectively promote stem/progenitor cell mobilization and recruitment while also suppressing expression of numerous members of the proinflammatory nuclear factor κB pathway, including myeloid differentiation primary response gene 88 and toll-interacting protein. Thus HOXA3 accelerates wound repair by mobilizing endothelial progenitor cells and attenuating the excessive inflammatory response of chronic wounds.

## INTRODUCTION

Tissue repair and regeneration require dramatic and coordinated changes in cell behavior in both wound-resident cells at the site of injury and in distant cells that respond to and are recruited to the injured tissue. Cells within the skin, normally homeostatic, respond to injury by increasing expression of genes that induce mitosis, migration, and direct synthesis and remodeling the extracellular matrix. This tightly regulated process involves a “ramping up” of growth factor and cytokine release followed by adult stem/progenitor cell mobilization, migration, engraftment, and finally, differentiation in response to injury. Studies investigating the genes involved in this process have identified both transcription factors and ligand/receptor pairs that have previously been implicated in wound healing. Genes such as *Hif-1α,* *Hoxa3, Cxcl12/Cxcr4,* and *Vegf/Vegfr2* among others play a variety of roles in wound repair and regeneration [1–5]; however, much of the genetic control of adult stem and progenitor cell behavior during wound repair and regeneration is largely unknown.

Angiogenesis is the growth of new blood vessels from existing blood vessels, whereas vasculogenesis is the growth of new blood vessels from stem or progenitor cells that are presumably recruited from the bone marrow. Previously we demonstrated that the transcription factor, HOXA3, significantly promotes angiogenesis/vasculogenesis during tissue repair and regeneration and accelerates cutaneous wound healing in vivo [3]. In particular we demonstrated that transient gene transfer of *HOXA3* to skin wounds of diabetic mice resulted in the upregulation of target genes, such as *Plaur, Mmp14,* and *Cdc42,* that are involved in extracellular matrix remodeling and cell migration.

A number of recent studies have also suggested that recruitment of bone marrow-derived cells (BMDCs) can enhance tissue repair in ischemic and myocardial infarction models, as well as in cutaneous wounds (recently reviewed in Wu et al [6]). Both angiogenesis and vasculogenesis can be improved in response to the presence of BMDCs in the wound milieu (recently reviewed in Schatteman et al [7]). Specifically, recruitment of multipotent mesenchymal stromal cells (MSCs) and endothelial progenitor cells (EPCs) can dramatically enhance neovascularization, either through direct incorporation and differentiation in the neovasculature, or by functioning as perivascular support cells that produce additional factors that stimulate repair [8–10].

BMDCs are first mobilized from the bone marrow and enter circulation in response to signals from the wound. Growth factors and cytokines such as VEGF and SDF-1/CXCL12 have been strongly implicated as signals that mediate this response [5, 11]. However, BMDCs consist of a heterogeneous population that can play repressive as well as supportive roles during wound healing. Over the past few decades, studies on the effects of inflammation during wound repair overwhelmingly support a model in which too many inflammatory cells and/or their overstimulation can impede the healing process [12–14]. Although mild inflammation early in response to injury may enhance angiogenesis/vasculogenesis, prolonged and/or excessive presence of leukocytes, activated neutrophils, and macrophages in particular, are associated with chronic wounds that have severely impaired healing [15, 16], and may interfere with efficient neovascularization.

Because many Hox genes have been found to play an important role in the regulation of blood stem cell proliferation and differentiation (recently reviewed in Argiropoulos and Humphries [17]), and the Hox-3 paralog group in particular has been shown to promote neovascularization [3, 18, 19], we hypothesized that *HOXA3*-augmented diabetic wound healing may in part arise from its potential impact on BMDCs. This could occur via modulation of BMDC mobilization, recruitment, or differentiation of stem/progenitor cells, or any combination of these processes during wound repair and regeneration. Here we show that sustained expression of *HOXA3* in diabetic wounds is accompanied by specific transcriptional changes in the wound microenvironment that modulate both the quantity and type of BMDCs recruited to cutaneous wounds, accelerating tissue repair.

## MATERIALS AND METHODS

### Mouse Strains and Bone Marrow Chimeras

All animals used in this study were housed in the University of California San Francisco (UCSF) animal care facility, and the UCSF Committee on Animal Research approved all procedures on animals. Male diabetic *(db/db)* mice (B6.Cg-*m*^+/+^ *Lepr*^*db*^/J; Jackson Laboratory, Bar Harbor, ME, http://www.jax.org) and heterozygous *(db/+)* controls age 8-10 weeks were lethally irradiated (10 Gy). Bone marrow was harvested from 8- to 10-week-old female donor mice ubiquitously expressing enhanced green fluorescent protein (GFP) (C57BL/6-Tg(CAG-EGFP)1Osb/J; Jackson Laboratory) by flushing the femurs and tibiae with Dulbecco's modified Eagle's medium. Bone marrow was passed through a sterile 27-gauge needle and through a 70-μm nylon mesh cell strainer (BD Biosciences, San Diego, http://www.bdbiosciences.com) and rinsed once in phosphate-buffered saline (PBS), and viable cells were counted. Chimeras were generated by injecting 10^6^ viable cells into the retro-orbital plexus of the male *db/db* or *db/+* mice approximately 6 hours after irradiation. Mice were maintained on antibiotic-treated water (Ditrim, 5 ml/200 ml) for 2 weeks, and allowed 4 weeks for reconstitution before any further procedures were performed.

### Wound Model and Methylcellulose-Mediated Gene Transfer

Prior to wounding, blood glucose levels were measured after a 12-hour fast using a standard portable blood glucose meter and GFP chimerism was measured by flow cytometry as described below. After confirmation of hyperglycemia in *db/db* recipients and GFP chimerism in all recipients, mice were wounded by making an 8-mm full thickness excisional wound on the dorsum as described previously [3]. Skin removed from the wound at this time was used for day 0. For chimera studies, *db/db* mice were randomly grouped into experimental and control groups (*n* = 6-8 for each group), and heterozygous *(db/+)* control groups (*n* = 3-4) for each time point (day 0, day 7, and day 14). For bone marrow and peripheral blood wound response quantitative reverse-transcription polymerase chain reaction (qRT-PCR) experiments, two *db/+* and two *db/db* mice were used for each time point (day 0 and day 4). For microarray experiments, four *db/db* mice were used, split into two groups, one experimental (*n* = 2) and one control group (*n* = 2), with skin from all four mice pooled for two technical replicates at the day 0 time point. Two mice were used from each group at day 4 for a total of four biological replicates. In each study, experimental groups were treated with cytomegalovirus promoter-driven *CMV-HOXA3* expression plasmid and control groups were treated with vector control. Plasmids were prepared as described previously [3], using 25 μg of DNA per 0.8-cm diameter wound, mixed, and dried down in 1% methyl cellulose to form a flat pellet. Twenty-five micrograms was determined as the optimal amount of plasmid DNA to achieve maximal expression, while minimizing toxicity, using a dose curve analysis of 5, 15, 25, 35, and 50 μg, Western blot quantification and qualitative wound healing analysis. Plasmid pellets were applied directly to the open wound at the time of wounding. Animals were housed individually and wounds left undressed. Mice were anesthetized prior to wounding with isoflurane gas (2%) in oxygen, and received 0.05 mg/kg buprenorphine subcutaneously at the time of wounding followed by a second dose 12 hours later.

### Immunohistochemistry

For the chimera studies, wounds were harvested at days 0, 7, and 14, incubated in formalin for 3 days, and embedded in paraffin. Five-μm sections were cut, dried for at least 24 hours, and stored at room temperature or processed immediately. Sections were deparaffinized in three changes of xylenes, followed by ethanol and methanol washes, and then rehydrated. Antigen retrieval was performed by microwaving sections in sodium citrate buffer (10 mM Na citrate, 0.05% Tween 20, pH 6.0) for 20 minutes, followed by standard immunofluorescent detection. Rabbit anti-GFP (Abcam, Cambridge, U.K., http://www.abcam.com) was used at a dilution of 1:1000; rat anti-Cd45 (Invitrogen, Carlsbad, CA, http://www.invitrogen.com), at 1:50; goat anti-Cd34 (Santa Cruz Biotechnology Inc., Santa Cruz, CA, http://www.scbt.com), at 1:100; rabbit anti-Cd29 (Abcam), at 1:250; and chicken anti-GFP (Abcam), at 1:500. Primary antibodies were detected with combinations of the following secondaries at 1:400: donkey anti-rabbit Alexa555 or goat anti-rabbit Alexa 488, donkey anti-rat Alexa 488 or goat anti-rat Alexa 555, donkey anti-goat Cy5, and goat anti-chicken Alexa 647, as appropriate.

### Flow Cytometry

Blood samples were analyzed from chimeras prior to wounding (GFP only) and on days 0, 4, 7, and 14, as follows: ∼200 μl of blood from each mouse was collected using a cheek lancet (Goldenrod; Medipoint, Minneola, NY, http://www.medipoint.com) into 50 μl of heparin solution, followed by ammonium chloride red blood cell (RBC) lysis and removal by centrifugation. Cleared blood was then analyzed by flow cytometry (LSR-II; BD Biosciences) and accompanying DIVA software (BD Biosciences) and FlowJo software (TreeStar, Inc., Ashland, OR, http://www.treestar.com). Samples were incubated with the following antibodies: anti-Cd34 Pacific Blue (1:20), anti-Cd133 PE (1:50), anti-Cxcr4 APC (1:20), anti-Cd45 PE-Cy7 (1:2000), and anti-Cd117 PE-Cy5.5 (1:200) (eBioscience, San Diego, http://www.ebioscience.com).

### RNA Isolation and Quantitative RT-PCR

Murine skin/wounds were harvested at the specified time points indicated and flash-frozen on dry ice and stored at −80° C or stored in RNA later (Ambion, Austin, TX, http://www.ambion.com) at 4° C. Tissue was then briefly rinsed in RNAse-free PBS (Ambion). RNA was isolated by tissue homogenization in Trizol (Invitrogen) with a rotor-stater homogenizer (Omni International, Marietta, GA, http://www.omni-inc.com). RNA was analyzed using a UV spectrophotometer and gel electrophoresis and stored at −80°C. One μg of total RNA was used in reverse transcription reactions using random primers in a total volume of 25 μl. qRT-PCRs were performed at least three times on each sample using approximately 25 ng of template cDNA, Taqman probe/primer pairs (*Hoxa3, Ccl2,* β-defensin *[Defb15], Myd88, Tollip,* and *Histone2A* & *18S* as reference controls), and universal master mix for each gene analyzed (Applied BioSystems, Foster City, CA, http://www. appliedbiosystems.com) on an ABI Prism SDS 7000 or Step-One-Plus machine according to the manufacturer's instructions. Data were analyzed with SDS 7000 or Step-One-Plus companion software and Microsoft Excel (Microsoft, Redmond, WA, http://www.microsoft.com).

### Microarray Analysis and Validation

Total RNA harvested from day 0, 4, and 7 wounds and universal mouse reference RNA (Stratagene, La Jolla, CA, http://www.stratagene.com) were amplified and labeled using the Amino Allyl MessageAmp II kit with Cy5 and Cy3 dUTP (Ambion). Labeled RNAs were then hybridized to murine whole genome expression (MEEBO) arrays (Stanford University). Arrays were scanned and analyzed using Stanford functional genomics facility scanner and accompanying software (Agilent Technologies, Palo Alto, CA, http://www.agilent.com). All arrays were inspected for hybridization defects using MEEBO-specific quality plots from the Bioconductor package arrayQuality [20], and print-tip loess normalization was used to correct for any dye or spatial biases [21]. Differentially expressed genes were determined using a Student's *t* test (*p* < .05), followed by additional intensity and fold-change filtering (log-intensity > 8 and absolute fold-change > 1.5). Differentially expressed genes were further analyzed for functional groups using WebGestalt (http://bioinfo. vanderbilt.edu/webgestalt) [22]. The minimum number of overrepresented genes was set to 3, and a *p* value cutoff of .05 was used for Kyoto Encyclopedia of Genes and Genomes (KEGG) pathway and Gene Ontology (GO) process identification. Complete data files are available at National Center for Biotechnology Information's Gene Expression Omnibus [23] http://www.ncbi.nlm.nih.gov/geo/query/acc.cgi?acc=GSE13324. Genes selected for validation and further analyses were quantified using qRT-PCR on RNA from microarray samples and at least two other samples collected independently.

### Statistical Analyses

Statistical significance was determined using a Student's *t* test for experiments where control and experimental groups were being compared, as noted in figures or text. Statistical significance was determined using analysis of variance, followed by a post hoc Tukey's test when more than two groups were being compared. A *p* value of < .05 was considered significant, unless otherwise noted.

## RESULTS

### Recruitment of BMDCs to Wounds Is Significantly Increased in Diabetic Mice

**Figure 1:**
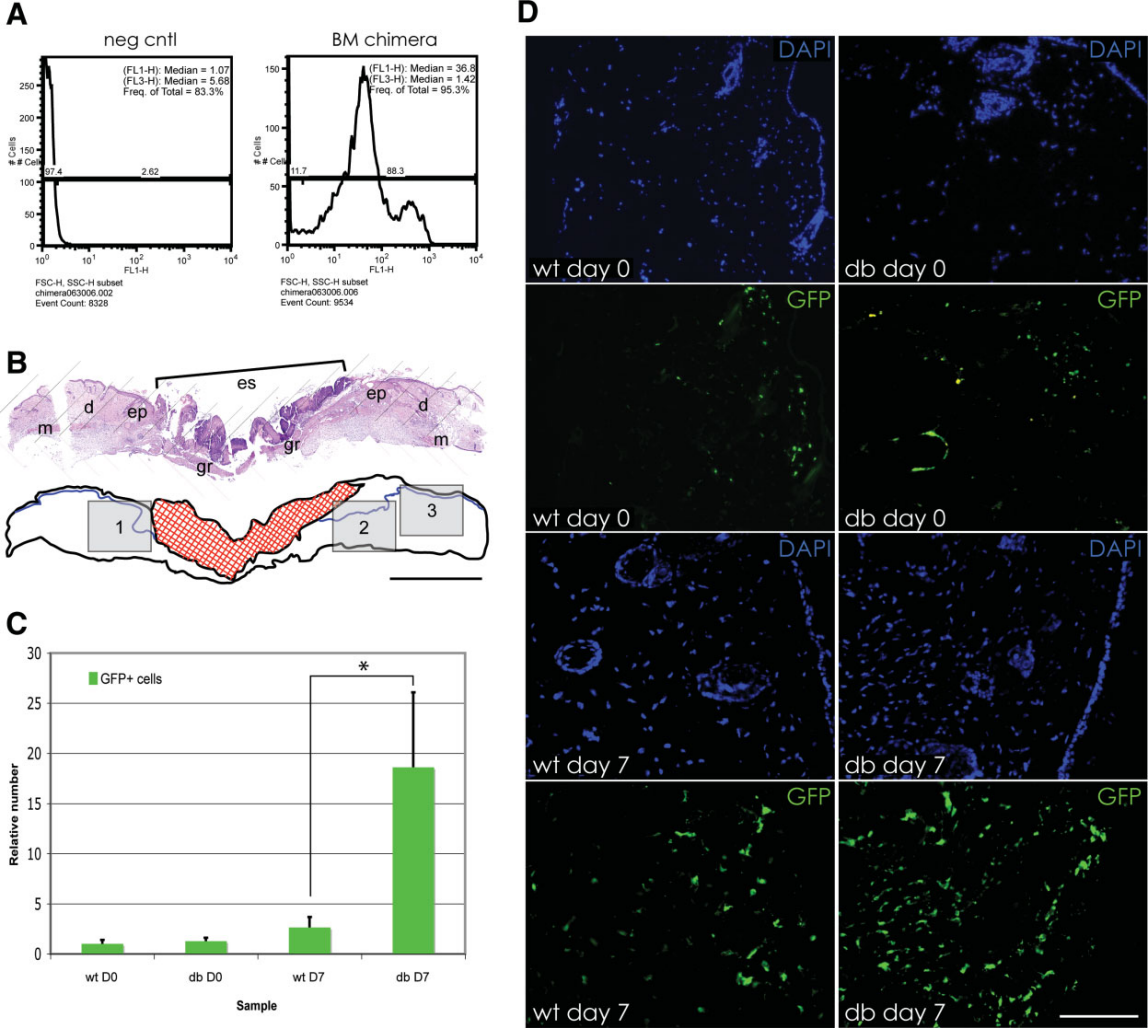
Bone marrow-derived cell (BMDC) recruitment to diabetic wounds in GFP bone marrow chimeras is dramatically increased compared with nondiabetic wounds. **(A):** Representative bone marrow chimerism flow cytometry analysis of peripheral blood using non-GFP chimera siblings as negative controls. **(B):** Representative day 7 wound section stained with hematoxylin and eosin is shown in the upper half of the panel. A graphic of this section is shown in the lower half of the panel. Red cross-hatching represents the wound. Rectangles 1, 2, and 3 represent areas near the wound boundary imaged using confocal microscopy to obtain average recruited BMDC number for each animal. Scale bar = 1 mm. **(C):** Relative number of BMDCs (normalized using DAPI-stained nuclei to account for cell density differences) recruited to the wound site in unwounded nondiabetic controls (wt D0), unwounded diabetic (db D0), day 7 wounds from nondiabetic (wt D7) and diabetic (db D7) mice. Asterisk indicates *p* < .05. **(D):** Representative images of DAPI staining and GFP immunofluorescence of BMDCs in wound sections as shown in **(B)** from nondiabetic (wt) and diabetic (db) mice at day 0 and day 7 following wounding. Scale bar = 100 μm. Abbreviations: d, dermis, DAPI, 4′,6-diamidino-2-phenylindole; db, diabetic; D0, day 0; D7, day 7; ep, epithelium, es, eschar, Freq, frequency; FSC-H, forward scatter height; GFP, green fluorescent protein; gr, granulation tissue; m, muscle; neg cntl, negative control; SSC-H, side scatter height; wt, wild type.

Diabetic wounds in both rodent models and human patients exhibit increased inflammation [24, 25]. However, recruitment of total BMDCs to diabetic wounds, which contain stem/progenitor cell types as well as inflammatory precursors, has not been previously analyzed, although addition of mononuclear bone marrow cells to diabetic wounds has been shown to improve healing [26]. To compare recruitment of BMDCs in diabetic and nondiabetic mice, we reconstituted lethally irradiated mice (6 *Lepr*^*db*−/−^ *“db/db”* diabetic mice and 4 *Lepr*^*db*+/−^ *“db/+”* nondiabetic littermate controls) with bone marrow from donor mice ubiquitously expressing GFP driven by the chicken β-actin promoter [27]. After 4 weeks of reconstitution, chimerism was analyzed by flow cytometry (Fig. 1A), with the mean percentage of GFP-positive cells in RBC-cleared blood greater than 80% (82.07 ± 4.05 SEM). Sections through unwounded skin (day 0) and 8-mm full thickness excisional wounds (day 7) of GFP chimeras were analyzed with hematoxylin and eosin to obtain similar positions within the wound tissue for further analysis. The day 7 time point was selected as it represents the peak of angiogenesis, and is well after the peak of the inflammatory phase, which occurs at days 3-4. Parallel sections were then analyzed for GFP^+^ cells, relative to 4′,6-diamidino-2-phenylindole (DAPI)-positive cells, in three separate areas near the wound edge to obtain an average for each wound (Fig. 1B). Although there is no significant difference in BMDC numbers in the skin of diabetic and nondiabetic mice prior to injury (day 0), there is a dramatic increase in total BMDC wound recruitment to cutaneous wounds by 7 days after injury. Nondiabetic mice exhibit an ∼3-fold increase in BMDCs in day 7 wounds compared with day 0, whereas diabetic mice exhibit an ∼18-fold increase in BMDCs in day 7 wounds compared with day 0 (Fig. 1C, 1D).

### Sustained Expression of *HOXA3* in Diabetic Wounds Reduces the Number of Recruited BMDCs

**Figure 2:**
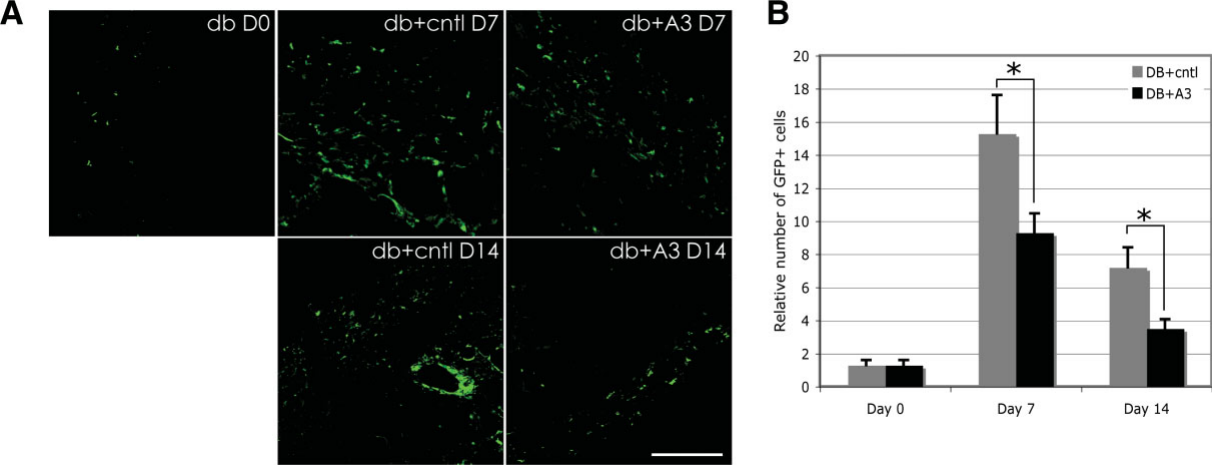
Expression of *HOXA3* during diabetic wound repair modulates the recruitment of bone marrow-derived cells (BMDCs). **(A):** Representative images of wound sections from diabetic mice with GFP immunofluorescent detection of BMDCs at days 0, 7, and 14 following wounding, treated with control (empty vector) or *CMV-HOXA3* plasmid. Scale bar = 100 μm. **(B):** Relative number of BMDCs (normalized against 4′,6-diamidino-2-phenylindole [DAPI]-stained nuclei) in wounds from diabetic mice treated with control (DB+cntl) or *CMV-HOXA3* plasmid (DB+A3) at day 0, day 7, and day 14 following wounding. Asterisk indicates *p* < .05. Abbreviations: DB+A3, diabetic wound treated with *CMV-HOXA3*; db+A3 D7, diabetic wound treated with *CMV-HOXA3* at day 7; db+A3 D14, diabetic wound treated with *CMV-HOXA3* at day 14; DB+cntl, diabetic wound treated with control; db+cntl D7, diabetic wound treated with control at day 7; db+cntl D14, diabetic wound treated with control at day 14; db D0, diabetic wound at day 0; GFP, green fluorescent protein.

As we previously demonstrated an increase in neovascularization due to wound-specific expression of *CMV-HOXA3* [3], we wished to test whether HOXA3 had any effect on recruitment of BMDCs, which are known to play a role in this process. We used GFP^+^ bone marrow chimeras to enumerate and identify BMDCs at different time points during the repair process. Three groups of mice were wounded with 8-mm-diameter full thickness excisional wounds as described previously [3]: 6 diabetic *(db/db)* mice were treated with 25 μg *CMV-HOXA3* plasmid; 6 diabetic mice were treated with 25 μg pcDNA3.1myc/his (empty vector), and 4 nondiabetic *(db/+)* mice were treated with 25 μg pcDNA3.1myc/his (empty vector), as this amount was determined to be optimal (see Materials and Methods for details). Nondiabetic mice were not treated with *CMV-HOXA3* as this has no observable effect on wound healing [3]. Immunolocalization of total GFP^+^ cells revealed that at days 7 and 14 after wounding the number of GFP^+^ cells in *db/db* mice treated with *CMV-HOXA3* was significantly reduced compared with vector control-treated mice (Fig. 2A, 2B).

### HOXA3 Suppresses Leukocyte Recruitment to Diabetic Wounds

**Figure 3:**
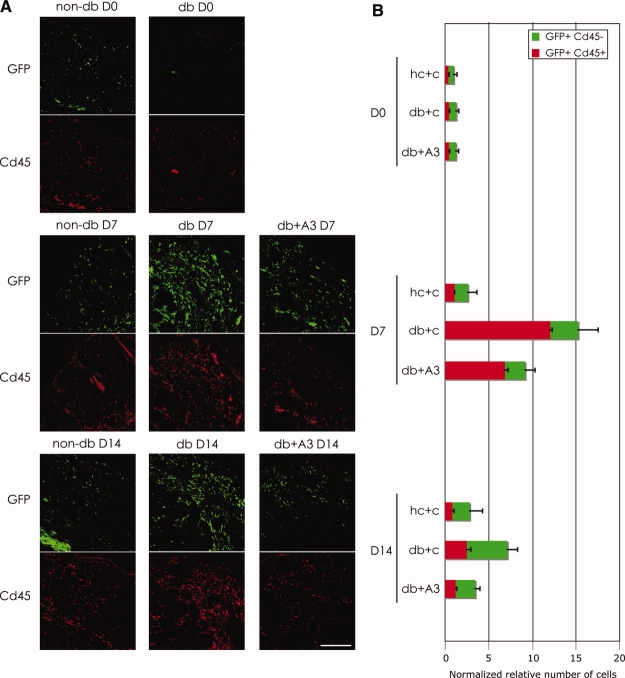
Expression of *HOXA3* in diabetic wounds suppresses inflammatory cell recruitment. **(A):** Representative images of sections from day 0, 7, and 14 wounds from nondiabetic and diabetic mice treated with control or *CMV-HOXA3* plasmid. Upper panels of each group show GFP^+^ cells and lower panels show Cd45^+^ cells. Scale bar = 100 μm. **(B):** Bar graphs showing normalized relative numbers of GFP^+^ and GFP^+^Cd45^+^ (leukocyte) cells recruited to the wounds at days 0, 7, and 14 in nondiabetic and diabetic mice treated with control or *CMV-HOXA3* plasmid. Abbreviations: D0, day 0; D7, day 7; D14, day 14; db+A3, diabetic wound treated with *CMV-HOXA3*; db+A3 D7, diabetic wound treated with *CMV-HOXA3* at day 7; db+A3 D14, diabetic wound treated with *CMV-HOXA3* at day 14; db+C and db+c, diabetic wound treated with control; db D0, diabetic wound at day 0; db D7, diabetic wound at day 7; db D14, diabetic wound at day 14; GFP, green fluorescent protein; hc+c, heterozygous control + control plasmid; non-db D0, nondiabetic wound at day 0; non-db D7, nondiabetic wound at day 7; non-db D14, nondiabetic wound at day 14.

Because the diabetic wound environment exhibits poor tissue repair, and is associated with chronic inflammation, we hypothesized that a larger percentage of the BMDCs recruited to these wounds at day 7 were leukocytes, compared with nondiabetic mice. Not surprisingly, as shown in Figure 3, double labeling of GFP and Cd45 (a pan-leukocyte marker) revealed that although diabetic mice have similar numbers of inflammatory cells at day 0, they showed a fivefold increase in GFP^+^Cd45^+^ leukocytes in day 7 wounds and a twofold increase in day 14 wounds compared with nondiabetic controls (Fig. 3A, 3B). This is consistent with previous reports that demonstrate both increased numbers and retention time of inflammatory cells in skin after injury in diabetic mice compared with nondiabetic mice [15, 16].

Furthermore, based on our results demonstrating that *HOXA3* gene transfer resulted in an overall reduction in the number of BMDCs, we wished to test whether sustained expression of *HOXA3* might result in a reduction in the number of leukocytes recruited to the wound, and perhaps promote healing by suppressing the chronic inflammation associated with diabetic wounds. We found *CMV-HOXA3* treatment significantly reduced the number of GFP^+^Cd45^+^ leukocytes present in the wound area at days 7 and 14 after wounding compared with empty vector control-treated mice (Fig. 3A, 3B). However, flow cytometry analysis of cleared (RBC-lysed) peripheral blood at days 0, 4, and 7 revealed that there was no significant difference in circulating GFP^+^Cd45^+^ cells at any of the assayed time points (data not shown), suggesting that the reduction of leukocytes in the wounds of *HOXA3*-treated diabetic mice was due to specifically suppressing leukocyte recruitment to the wound or retention at the wound site, rather than a reduction in mobilization from the bone marrow.

### *HOXA3* Expression in Diabetic Wounds Stimulates Bone Marrow Mobilization and Recruitment of EPCs

**Figure 4:**
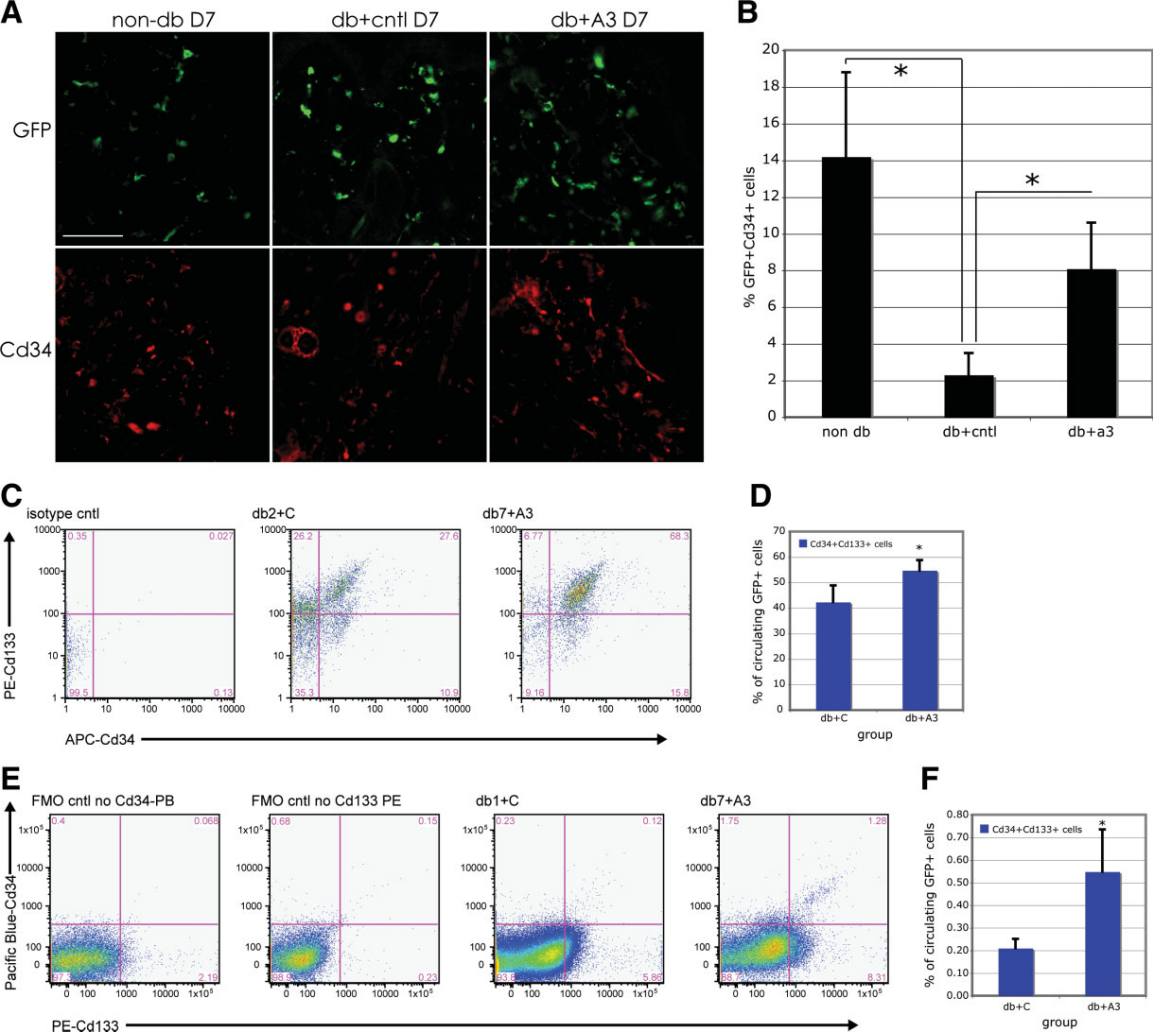
Expression of *HOXA3* in diabetic wounds stimulates mobilization of endothelial progenitor cells (EPCs). **(A):** Representative images of wound sections from nondiabetic mice (non-db) and diabetic mice (db) treated with control (empty vector) or *CMV-HOXA3* plasmid. Upper panels show GFP^+^ bone marrow-derived cells at day 7 and lower panels show Cd34^+^ cells. Scale bar = 100 μm. **(B):** Percentage of total GFP^+^ cells that are double positive for Cd34 recruited to the wound at day 7 in nondiabetic mice treated with control plasmid, and diabetic mice treated with control (db+cntl) or *CMV-HOXA3* (db+A3) plasmid. **(C):** Representative scatter plots of cleared peripheral blood from diabetic GFP chimeras at day 4 following wounding treated with control (db2+c) or *CMV-HOXA3* (db7+A3) plasmid. Cells in the upper right quadrant are GFP^+^Cd133^+^Cd34^+^ (early EPC) cells. **(D):** Quantification of early EPCs in peripheral blood at day 4 following wounding of control-treated or *CMV-HOXA3*-treated diabetic wounds. **(E):** Representative scatter plots of cleared peripheral blood from diabetic GFP chimeras at day 7 following wounding treated with control (db2+c) or *CMV-HOXA3* (db7+A3) plasmid. Cells in the upper right quadrant are GFP^+^Cd133^+^Cd34^+^ (early EPC) cells. **(F):** Quantification of early EPCs in peripheral blood at day 7 following wounding of control-treated or *CMV-HOXA3*-treated diabetic wounds. Abbreviations: APC, allophycocyanin; db+A3 D7, diabetic wound treated with *CMV-HOXA3* at day 7; db+a3 and db+A3, diabetic wound treated with *CMV-HOXA3* at day 3; db+C, diabetic wound treated with control; db+cntl, diabetic wound treated with control; db+cntl D7, diabetic wound treated with control at day 7; db1+C, diabetic wound 1 treated with control; db2+C, diabetic wound 2 treated with control; db7+A3, diabetic wound treated with *CMV-HOXA3* at day 7; FMO, fluorescence minus one control; GFP, green fluorescent protein; non-db D7, nondiabetic wound at day 7; non db, nondiabetic wound; PB, peripheral blood; PE, phycoerythrin.

Because HOXA3 strongly stimulates neovascularization in the wound [3], we also investigated whether EPC recruitment to the wound was influenced. Immunolocalization of EPCs was performed using double and/or triple labeling of antibodies against GFP, Cd133, and Cd34. The antibody combination detecting GFP, Cd133, and Cd34 marks a population of very early EPCs, whereas the GFP^+^Cd34^+^Cd133^−^ cells are regarded as more mature EPCs [28, 29]. Despite the reduction in total BMDCs and Cd45^+^ cells shown in Figure 3, *db/db* mice treated with *CMV-HOXA3* displayed a significant increase in GFP^+^Cd34^+^ EPCs in wounds at day 7 compared with vector control-treated *db/db* (Fig. 4A, 4B). This relative increase was still present at day 14, although to a much reduced extent (not shown). Cd133 was not detectable in the wound tissue, suggesting that cells retained in the wound were more mature EPCs. Additionally, because multipotent MSCs can express Cd34 and can also be recruited from the bone marrow, as well as surrounding adipose tissue, and contribute to angiogenesis we could not rule out that some of these cells were MSCs as opposed to “classic” EPCs [30]. To detect non-BM-derived MSCs, we used a combination of Cd29 (integrin β-1), which murine MSCs consistently express, Cd45, which MSCs consistently do not express [30], and GFP to mark cells derived from the bone marrow (this was also negatively selected against). We found no difference in non-BM-derived MSC recruitment between *HOXA3*-treated and control-treated wounds (data not shown).

To test whether the differences in EPCs present in *HOXA3*-treated wounds at day 7 were due to enhanced EPC recruitment, and/or could be attributed to increased mobilization of EPCs from the bone marrow, we analyzed blood samples collected both from *db/db* mice treated with *CMV-HOXA3* or control plasmid, as well as *db/+* mice treated with control plasmid. Flow cytometry was performed to analyze cleared peripheral blood samples using GFP, Cd34, and Cd133 as markers of EPCs at the following time points: day 0, day 4, day 7, and day 14. Although there were no significant differences in GFP^+^Cd34^+^Cd133^−^ cells (more mature EPCs), “early” EPCs (GFP^+^Cd34^+^Cd133^+^ cells) showed a significant increase in circulation in *CMV-HOXA3*-treated *db/db* mice compared with controls at days 4 and 7 after injury (Fig. 4C--4F). Thus HOXA3 functions not only to reduce inflammatory cell infiltration into wounds, but also to selectively mobilize and recruit EPCs, and possibly BM-derived Cd34^+^ MSCs to wounds, promoting healing.

### Analysis of Cell-Specific Transgene Expression

**Figure 5:**
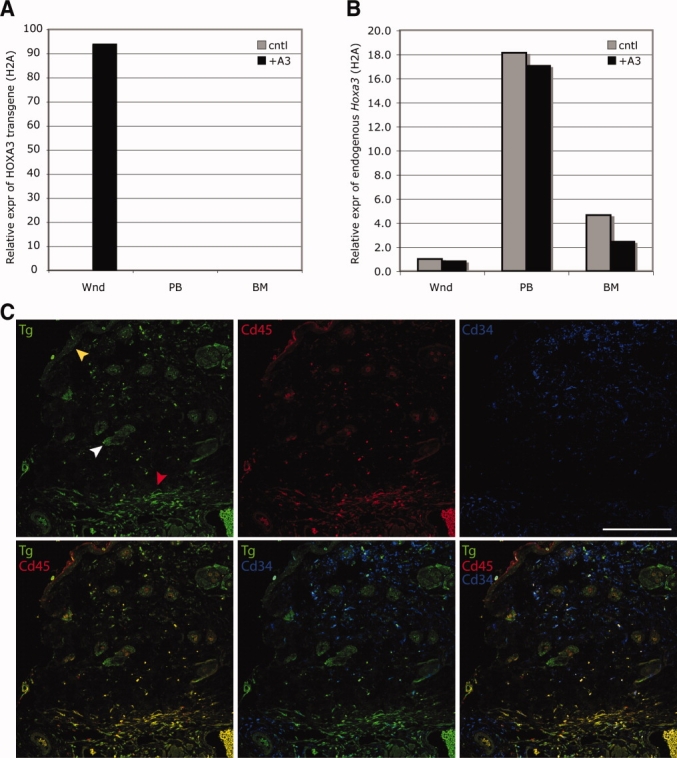
The *HOXA3* transgene is not expressed in peripheral blood or bone marrow cells and has no effect on endogenous *Hoxa3*. **(A):** Quantitative reverse-transcription polymerase chain reaction (RT-PCR) of RNA isolated from wound tissue, peripheral blood, and bone marrow from diabetic mice treated with control or *CMV-HOXA3* plasmid showing relative expression of the *HOXA3* transgene at day 4 following wounding. **(B):** Quantitative RT-PCR of RNA isolated from wound tissue from diabetic mice treated with control or *CMV-HOXA3* plasmid showing relative expression of endogenous *Hoxa3*. **(C):** Analysis of transgene expression 4 days after wounding and gene transfer. Transgene expression is shown in green in the upper left panel, white arrowhead indicates expression in hair follicle cells, yellow arrowhead indicates expression in basal keratinocytes, and red arrowhead indicates expression in leukocytes. Leukocytes are marked with red (Cd45) in upper middle panel, and Cd34^+^ cells are shown blue in upper right panel. Lower panels show merged channels as indicated. Scale bar = 100 μm. Abbreviations: +A3, plus *HOXA3;* BM, bone marrow; cntl, control; PB, peripheral blood; Tg, transgene; Wnd, wound tissue.

Because changes in Hox gene expression have been shown to have dramatic effects on bone marrow-derived progenitor cells, including increasing proliferation and self-renewal in the stem cell compartment (recently reviewed in Argiropoulos and Humphries [17]), we evaluated whether local gene transfer of *HOXA3* to wounds could also result in transfection of circulating cells or bone marrow-resident cells, and thus alter their fate. Quantitative PCR analyses using primer sets specific to the human *HOXA3* transgene revealed that although the *HOXA3* transgene was easily detectable in wound tissue 4 days after gene transfer, it was not detectable in peripheral blood or bone marrow (Fig. 5A). Accordingly, levels of endogenous *Hoxa3* expression were similar in circulating cells and bone marrow cells of *HOXA3*-treated and vector control-treated animals (Fig. 5B). We next investigated whether recruited BMDCs, in particular leukocytes (Cd45^+^) and/or EPCs (Cd34^+^) cells, were transfected by application of transgene to the wound, thus affecting their behavior within the wound. To avoid problems with detection of endogenous Hoxa3, we performed a gene transfer experiment using CMV-GFP. As shown in Figure 5C, expression of the transgene in wounds at day 4 is overwhelmingly biased toward leukocytes, suggesting that the largest effect of *HOXA3* gene transfer to wounds is due to direct HOXA3-mediated changes in leukocyte behavior.

### Microarray Analyses of HOXA3-Mediated Changes in Wound Tissue Gene Expression

**Figure 6:**
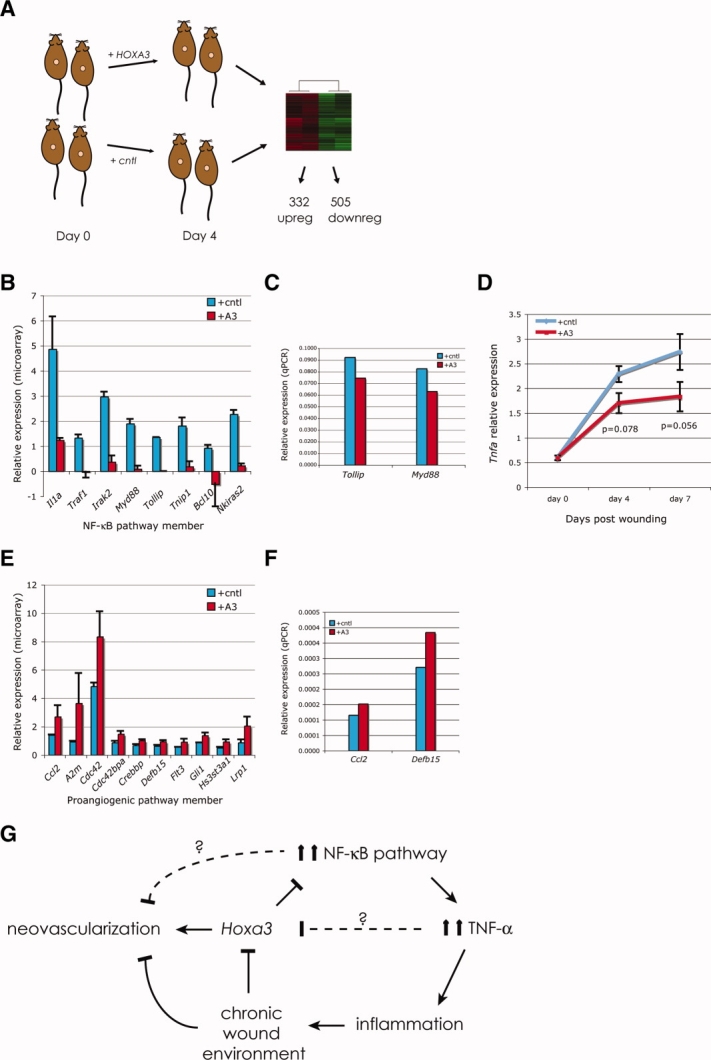
Expression of *HOXA3* in diabetic wounds results in activation of proangiogenic genes and repression of proinflammatory genes at the wound site. **(A):** Schematic representation of microarray experiment. Mice were split into two groups; unwounded skin was harvested at day 0 and wounded skin, at day 4. Gene expression profiles of control-treated and *HOXA3*-treated wounds were compared. **(B):** NF-κB pathway member gene expression analysis in response to *CMV-HOXA3* treatment. **(C):** Quantitative reverse-transcription polymerase chain reaction (qRT-PCR) validation of selected genes that are central to NF-κB pathway signal transduction. **(D):** qRT-PCR of *Tnfa* expression in control-treated and *CMV-HOXA3*-treated wounds at days 0, 4, and 7. **(E):** Proangiogenic gene expression analysis in response to *CMV-HOXA3* treatment of diabetic wounds. **(F):** qRT-PCR validation of selected genes known to promote endothelial progenitor cell (EPC) mobilization and recruitment to sites of neovascularization. **(G):** Model of HOXA3 function during wound repair. Expression of *HOXA3* in diabetic wounds results in downregulation of members of the NF-κB pathway, leading to a decrease in *Tnf-α* expression. This results in reduced inflammation and a wound environment more conducive to repair and vascular regeneration. Abbreviations: +A3, plus *HOXA3*; cntl, control; downreg, downregulation; NF-κB, nuclear factor κB; qPCR, quantitative polymerase chain reaction; TNF-α, tumor necrosis factor α; upreg, upregulation.

To investigate how HOXA3 expression in wound tissue alters gene expression to control selective recruitment of BMDC populations, we used MEEBO arrays (see Materials and Methods) to identify differentially expressed genes in cutaneous wounds from *db/db* mice treated with either *CMV-HOXA3* or control plasmid. We chose day 4 as the time point to harvest the wounds for RNA isolation, as this day represents the transition from inflammatory to proliferative phase and precedes the observed differences in BMDC mobilization and recruitment at day 7 (Fig. 6A).

Day 4 wounds treated with *CMV-HOXA3* showed significant differential expression of 837 known genes by at least 1.5-fold (*p* < .05), with 505 genes downregulated and 332 genes upregulated in response to *CMV-HOXA3* treatment (Fig. 6A, supporting information data 1 and 2; complete data files have been deposited at http://www.ncbi.nlm.nih.gov/geo/query/acc.cgi?acc=GSE13324). Analyses of the HOXA3-repressed gene set revealed regulatory pathways inhibited by HOXA3. Seven of these pathways are specific to leukocyte signaling and differentiation (Table 1, left-hand column, highlighted pathways), consistent with our findings that HOXA3 suppresses the inflammatory response. In particular, many members of the nuclear factor κB (NF-κB) pathway were repressed, including interleukin 1 alpha, Tnf receptor-associated factor 1, interleukin-1 receptor-associated kinase 2, myeloid differentiation primary response gene 88, toll-interacting protein, TNFAIP3-interacting protein 1, B-cell leukemia/lymphoma 10, and NF-κB inhibitor-interacting Ras-like protein 2 (Fig. 6B). Because of their central role in NF-κB signaling in inflammatory cells, *Myd88* and *Tollip* were selected for further analysis and validated using qRT-PCR (Fig. 6C). We also analyzed *Tnf-α* expression, as it is one of the major targets of NF-κB activation. Not surprisingly, mice treated with *HOXA3* showed reduced *Tnf-α* transcriptional activation at both day 4 (*p* = .078) and day 7 (*p* = .056) (Fig. 6D). This HOXA3-mediated repression of proinflammatory genes at day 4 is not due to a decrease in the number of inflammatory cells present in the wound at day 4, as the numbers of GFP^+^Cd45^+^ cells in the wound at day 4 did not significantly differ between *HOXA3*-treated and control-treated *db/db* wounds at this time point (data not shown). All together, these data suggest that HOXA3 suppresses the exacerbated inflammatory cell response found in diabetic chronic wounds in part by silencing the NF-κB pathway in cells in the wound by day 4, resulting in a significant reduction of inflammatory cells in the wound by day 7, coinciding with the peak of the neovascularization phase.

**1:** Regulatory pathways modulated by HOXA3 during wound repair

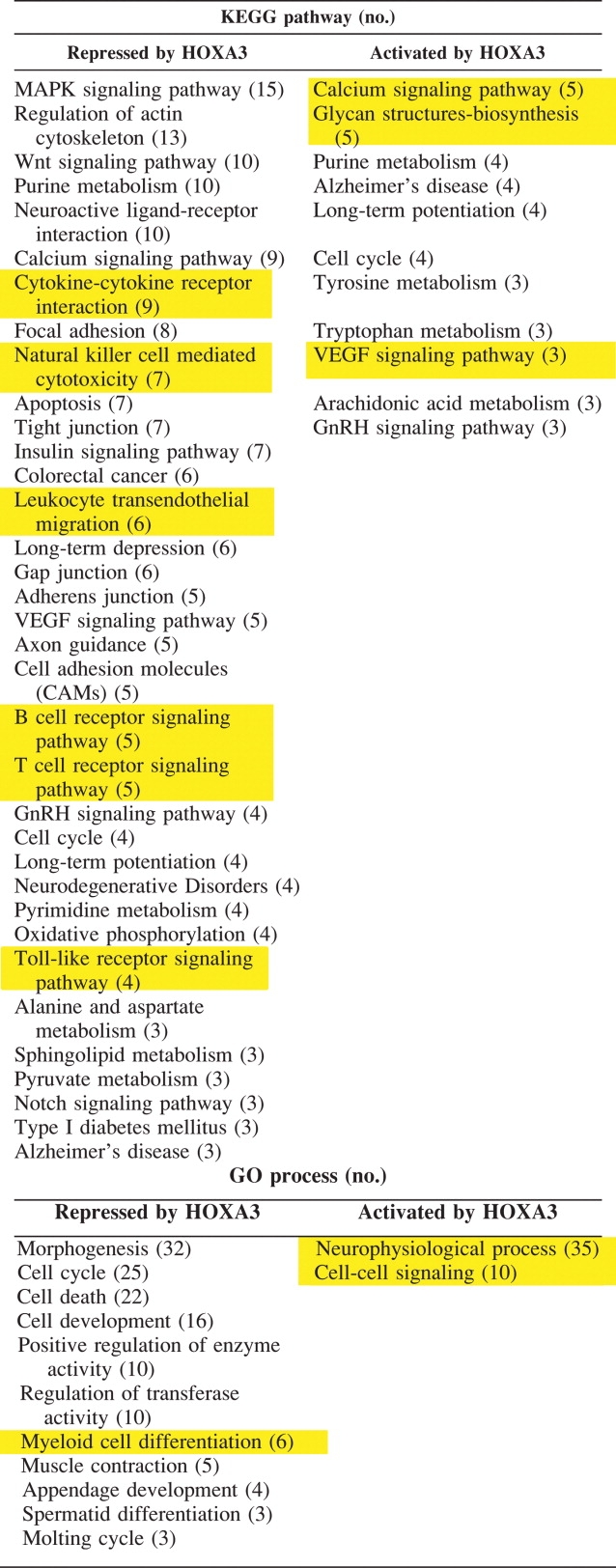

Analyses of the much smaller set of upregulated genes in *db/db* wounds at day 4 did not reveal any KEGG pathways that are specific to neovascularization, per se, however several of the pathways promoted by HOXA3 are known to contribute to neovascularization, and in particular, to endothelial cell migration (Table 1, right column, highlighted in yellow), such as calcium signaling, glycan biosynthesis, and vascular endothelial growth factor (VEGF) signaling pathway components. GO function analyses of this gene set suggest HOXA3 promotes cell-cell signaling pathways and neurophysiological processes (Table 1, right column, highlighted in yellow), both of which have also been shown to play a significant role in neovascularization (reviewed in Lok et al [31] and Scott et al [32]). However, because HOXA3 has been previously shown to promote angiogenesis and endothelial cell migration, we chose to focus on factors from this gene set that directly promote EPC mobilization and EPC/endothelial cell migration (Fig. 6E). Although we did not detect any changes in *Vegf* expression, chemoattractant molecules monocyte chemoattractant protein-1 *(MCP-1/Ccl2)* and *Defb15,* both potent inducers of EPC mobilization and recruitment [33, 34], were independently confirmed by quantitative PCR (Fig. 6F). Interestingly, *Lrp1,* which is both proangiogenic [35, 36] and anti-inflammatory, via inhibition of the NF-κB pathway [37], was also increased (Fig. 6E).

All together, these data suggest that HOXA3 acts within wound tissue to increase expression of angiogenic and EPC recruiting factors, while also repressing expression of genes that contribute to inflammatory cell activation and recruitment, resulting in enhanced neovascularization and tissue repair. Suppression of the NF-κB pathway by HOXA3 results in a reduction of TNF-α and inflammation, which are associated with the chronic wound environment and reduced HOXA3 expression characteristic of diabetic wounds. This “rescue” of the chronic wound environment is, in turn, associated with enhanced neovascularization (Fig. 6G).

## DISCUSSION

In this study we have demonstrated that sustained expression of *HOXA3* in diabetic wounds significantly alters BMDC recruitment in response to wounding. We show that local expression of HOXA3 in wound tissue not only results in increased mobilization and recruitment of EPCs, but also suppresses the excessive inflammatory response characteristic of diabetic wounds. Together, the enhanced angiogenic response and reduced inflammation lead to a significant acceleration of healing.

Previous attempts to improve healing in diabetic wounds have focused either on improving angiogenesis via direct activation and/or enhancing recruitment of EPCs, or reducing excessive inflammation, and have met with various degrees of success [24, 38–40]. Reduced angiogenesis in diabetic wounds has been attributed to reduced mobilization, function, and engraftment of EPCs into tissues [39, 41]. However, direct application of SDF-1/CXCL12 enhances mobilization of EPCs and improves healing, specifically enhancing recruitment of Cd34^+^ EPCs into the neovasculature of granulation tissue [39, 42]. In the current study we have shown that HOXA3 induces expression of MCP-1/Ccl2, a related member of the CxC/CC chemokine family that has previously been shown to directly recruit EPCs [43] and stimulate angiogenesis [44]. Although we did not detect any differences in VEGF expression itself, reduced VEGF signaling, as well as aberrant cell adhesion properties of EPCs to endothelial cells in diabetic wounds, also contributes to poor EPC recruitment and retention [45–47]. VEGF itself has been shown sufficient for recruitment and retention of recruited bone marrow-derived circulating cells in wild type mice [5]. VEGF can accelerate wound healing when topically applied to diabetic wounds by promoting angiogenesis and recruitment of Tie2^+^ EPCs [47]. However, VEGF and other growth factor therapy in patients has proven less successful than expected, most likely due to high levels of proteases and advanced glycation end products (AGEs), both of which are associated with excessive inflammation in the diabetic wound environment [16, 48]. High levels of glucose can react nonenzymatically with the amino groups of proteins (glycation). One consequence of this is reduced effectiveness of downstream components of the VEGF signaling pathway [49]. Thus, treatment of damaged tissue, even with high levels of ligand, is ineffective. Furthermore, AGE-modified fibronectin significantly reduced EPC attachment [50], whereas blockade of the receptor for AGE significantly improved vascularization in diabetic mice [16]. Our findings that peripheral blood of *HOXA3*-treated animals contained greater numbers of immature Cd34^+^Cd133^+^ EPCs, whereas wounds contained predominantly more mature Cd34^+^Cd133^−^ EPCs, suggest that HOXA3 not only increased mobilization of immature EPCs but also created an appropriate wound environment that was permissive to adhesion and subsequent maturation of the recruited EPCs. This environment may have also stimulated additional stem/progenitor cells not derived from the bone marrow, and thus not detectable by our GFP tracking methods, as these studies are based on cells derived from reconstituted bone marrow cells descended from hematopoietic stem cells.

Chronic wounds also exhibit high levels of proteases that can modify the provisional extracellular matrix necessary for proper EPC adhesion [51, 52]. The high levels of proteases present in diabetic and chronic wounds have been linked to excessive inflammation with recruited neutrophils and macrophages expressing high levels of a variety of matrix-degrading proteins [15]. A role for limiting inflammation in improving tissue repair is also supported by studies in *Pu.1*-null mice that lack neutrophils and macrophages. These mice healed faster and displayed no evidence of fibrosis compared with their wild type counterparts [14]. Importantly, in diabetic wounds, neutralization of TNF-α activity resulted in improved repair and healing due to inactivation of macrophages [40], and our present results show that in addition to improving EPC recruitment, HOXA3 also attenuates the TNF pathway, reducing inflammation. Specifically we show that HOXA3 inhibits hyperactivation of the NF-κB pathway, resulting in attenuation of *Tnf-α* expression, and leading to an overall reduction in Cd45^+^ leukocytes within the diabetic wound tissue. *Myd88* and *Tollip,* both significantly downregulated by HOXA3 treatment, encode central components of the NF-κB pathway, functioning as adaptor proteins required for most Toll-like receptor activation of NF-κB in both wound-resident cells and recruited BMDCs. Interestingly, complete loss of *Myd88* function, however, results in impaired wound healing and poor neovascularization [53]. Thus it seems that the correct balance of NF-κB activation must be achieved for efficient repair and regeneration. Concentration-dependent differential gene regulation by activated NF-κB is well documented (reviewed in Stathopoulos and Levine [54] and Sur et al [55]). We speculate that high levels of nuclear NF-κB result in transcription of genes promoting recruitment/retention of leukocytes, whereas moderate levels of nuclear NF-κB do not, thus facilitating healing.

### Differential Recruitment of EPCs and Leukocytes?

The majority of EPCs as well as inflammatory cells recruited to the wound are derived form stem/progenitor populations within the bone marrow. However, it is not clear how different populations within the bone marrow are differentially mobilized. For example, MCP-1/Ccl2 can mobilize EPCs and monocyte populations. Although application of this factor has been linked to improved repair and increased EPC recruitment, *Ccl2*-null mice also displayed reduced inflammation and skin fibrosis after wounding [56]. Thus the precise mechanisms that allow for enhanced EPC recruitment but restrict leukocyte retention in *HOXA3*-treated wounds are not clear. Whether enhanced EPC recruitment accelerates vascular maturation and stability, which in turn would reduce leukocyte extravasation, is not known. One possibility is that attenuated NF-κB activity results in a switch in phenotype of the recruited monocytes, resulting in their differentiation into endothelial cells or endothelial support cells instead of activated macrophages. This idea is supported by studies demonstrating that hematopoietic progenitor cell differentiation into an activated dendritic cell can be blocked by VEGF receptor activation on the progenitor cell and requires inhibition of NF-κB [57]. Furthermore, environmental cues, such as continued proangiogenic stimulation by factors such as VEGF, promote the differentiation of monocytic progenitor cells into endothelial-like cells [58]. Future studies will be focused on elucidating the precise gene regulatory networks downstream of HOXA3 function controlling the recruitment and behavior of EPCs to sites of injury.

## SUMMARY

This study indicates that the ability of HOXA3 to activate multiple linked pathways to improve angiogenesis and EPC recruitment, as well as suppressing inflammatory pathways that can inhibit repair and regeneration, comprises a truly successful therapy for diabetic chronic wounds. HOXA3 acts as a “master control” transcription factor that sits at the top of a large transcriptional regulatory network, and affects a variety of pathways that regulate these processes in a developmental context [59]. It is tempting to speculate that restoring expression of “master morphoregulatory factors” important during embryonic development may significantly contribute to tissue repair therapies in adults, and perhaps provide a key to unlocking the potential for true tissue regeneration.

## DISCLOSURE OF POTENTIAL CONFLICTS OF INTEREST

The authors indicate no potential conflicts of interest.
